# Sensitivity and Resolution Improvement in RGBW Color Filter Array Sensor

**DOI:** 10.3390/s18051647

**Published:** 2018-05-21

**Authors:** Seunghoon Jee, Ki Sun Song, Moon Gi Kang

**Affiliations:** Department of Electrical and Electronic Engineering, Yonsei University, 50 Yonsei Road, Seodaemun-gu, Seoul 03722, Korea; jeedang1109@hanmail.net (S.J.); iamhoh@naver.com (K.S.S.)

**Keywords:** red-green-blue-white (RGBW), demosaicing, texture decomposition, color filter array, sensitivity improvement, resolution improvement

## Abstract

Recently, several red-green-blue-white (RGBW) color filter arrays (CFAs), which include highly sensitive W pixels, have been proposed. However, RGBW CFA patterns suffer from spatial resolution degradation owing to the sensor composition having more color components than the Bayer CFA pattern. RGBW CFA demosaicing methods reconstruct resolution using the correlation between white (W) pixels and pixels of other colors, which does not improve the red-green-blue (RGB) channel sensitivity to the W channel level. In this paper, we thus propose a demosaiced image post-processing method to improve the RGBW CFA sensitivity and resolution. The proposed method decomposes texture components containing image noise and resolution information. The RGB channel sensitivity and resolution are improved through updating the W channel texture component with those of RGB channels. For this process, a cross multilateral filter (CMF) is proposed. It decomposes the smoothness component from the texture component using color difference information and distinguishes color components through that information. Moreover, it decomposes texture components, luminance noise, color noise, and color aliasing artifacts from the demosaiced images. Finally, by updating the texture of the RGB channels with the W channel texture components, the proposed algorithm improves the sensitivity and resolution. Results show that the proposed method is effective, while maintaining W pixel resolution characteristics and improving sensitivity from the signal-to-noise ratio value by approximately 4.5 dB.

## 1. Introduction

**Figure 1:**
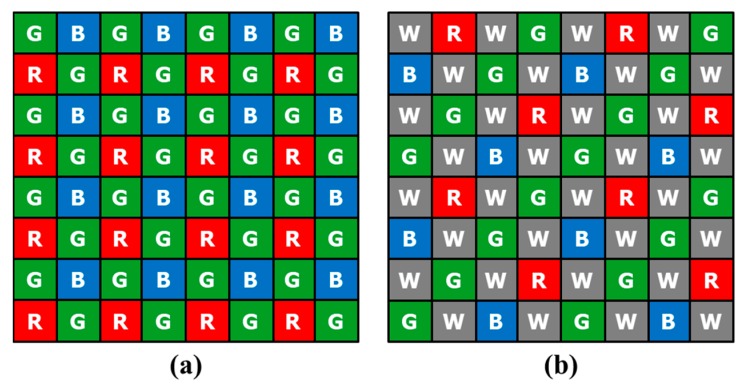
Color filter array (CFA) patterns: (**a**) Bayer [1]; (**b**) Sony red-green-blue-white (RGBW) [2].

Most digital imaging devices use a color filter array (CFA) to reduce the equipment cost and size instead of using three sensors and optical beam splitters. The Bayer CFA, which consists of the primary colors red, green, and blue (R, G, and B), is a widely used CFA pattern [1] and is shown in Figure 1a. Recently, methods using a new CFA have been studied to overcome the limited sensitivity of the Bayer CFA under low-light conditions. This is because the amount of absorbed light decreases on account of the RGB color filters. Hence, new CFA patterns [2,3,4,5] and demosaicing methods [6,7,8,9] have been proposed that contain panchromatic white (W) pixels in the pattern. Figure 1b depicts an example of a red-green-blue-white (RGBW) CFA named the Sony RGBW [2]. In this example, the W pixel has a much wider spectral band than the R, G, and B pixels; therefore, the W pixel can absorb more photons than other color pixels. For this reason, the W pixels are more robust to image noise.

Despite their improved sensitivity, various RGBW CFA patterns suffer from spatial resolution degradation. This is because the sensor is composed of more color components than the Bayer CFA pattern. Figure 1 shows the Bayer CFA and Sony RGBW CFA. In Figure 1b, 50% of the total area is occupied by the W channel, 25% by the G channel, and 12.5% by the R and B channels, respectively. In the demosaicing result of the RGBW CFA, the W channel resolution will be similar to the resolution of the G channel of the demosaicing result of the Bayer CFA image. Moreover, the resolution of the RGBW CFA G channel will be similar to that of the Bayer CFA R or B channel. Likewise, the resolution of the R and B channels of the RGBW CFA will be less than that of the R and B channels of the Bayer CFA. Consequently, various demosaicing methods have been proposed to overcome the RGBW CFA resolution problem. 

In conventional methods, the conversion from the acquired RGBW pattern to the color image is usually performed in two steps. First, the RGBW pattern is converted into the widely used Bayer CFA pattern. Second, the Bayer CFA pattern is converted into the color image. The RGBW pattern is first converted into the Bayer CFA pattern because the conversion is relatively easy and numerous demosaicing and denoising algorithms [10,11,12,13,14,15,16,17,18,19,20,21,22,23] exist for the Bayer CFA pattern. However, the reconstructed color image is degraded by this two-step conversion, since both steps introduce aliasing artifacts and color distortions, which aggravate the image when combined.

Another method is to modify the multiscale-gradient (MSG)-based demosaicing algorithm, which is commonly used in the Bayer CFA, into the Sony RGBW CFA [24]. The main assumption for MSG-based methods is that all color bands have spectral correlations and similar image structures, such as high-frequency components. This method is more effective with respect to the resolution than the two-step method because it performs demosaicing only once. In addition, since the high-frequency component of the W channel is shared with the R, G, and B channels, the resolution of the demosaiced image is improved compared with the conventional method. In addition, a demosaicing algorithm based on analysis of the frequency characteristics of various RGBW patterns was presented [25]. This method applies the demosaicing algorithm based on analysis of the frequency characteristics of the conventional RGB pattern [18]. Additionally, a pan-sharpening-based demosaicing method exists [26] that utilizes the resolution characteristics of W from a high density of W pixels. These demosaicing methods have been applied to various patterns in the existing Bayer CFA pattern and are one-step methods. Therefore, they show fewer aliasing artifacts than the two-step methods. However, because demosaicing algorithms are primarily intended to solve resolution and color-aliasing problems, the W high sensitivity (brightness and noise) characteristics are not reflected. Although demosaicing algorithms improve the sensitivity of the output image, on account of the influence of low noise in the process of estimating the W channel correlations in the resolution, they do not fully reflect the sensitivity of W channel.

In this paper, we therefore propose a post-processing method that can enhance the spatial resolution while improving the brightness and signal-to-noise ratio (SNR) of the RGB channels by reflecting the W channel sensitivity. Since many RGBW CFAs have a high density of W channels [2,3,5], demosaicing algorithms can reconstruct the W channel for high sensitivity and high resolution. On the other hand, the R, G, and B channels have a lower resolution due to their having a lower density than the Bayer CFA, which causes more intense color aliasing. For this reason, we propose a method to improve the resolution and SNR of R, G and B channels by updating the W channel texture component with consideration of the similarity of the texture components between channels.

The texture component is a kind of high-frequency component, except for the structural part, such as strong edges at object boundaries. The texture component includes noise and color aliasing artifacts, as well as image resolution. Since the proposed post-processing method is based on a texture decomposition technique, we propose a new texture separator. For the texture decomposer, we propose a cross multilateral filter (CMF), which is a kind of edge-preserving image-smoothing filter that is robust to color component edges for texture component separation. The proposed CMF extends the existing cross bilateral filter (CBF), which is a smoothing filter applied to the target image by estimating the filter kernel with a single guide image. The proposed CMF is robust to color component edges and effectively uses color channels, as well as W channels, as guide images to effectively suppress color aliasing and color noise.

The remainder of this paper is organized as follows. In Section 2, the research problem is detailed. In Section 3, the proposed demosaiced image post-processing method for RGBW CFA is described. In Section 4, we present the results of conducted experiments, and we compare the results of the proposed method with those of Bayer CFA demosaicing and RGBW CFA demosaicing. Our conclusions are presented in Section 5.

## 2. Problem Statement

**Figure 2:**
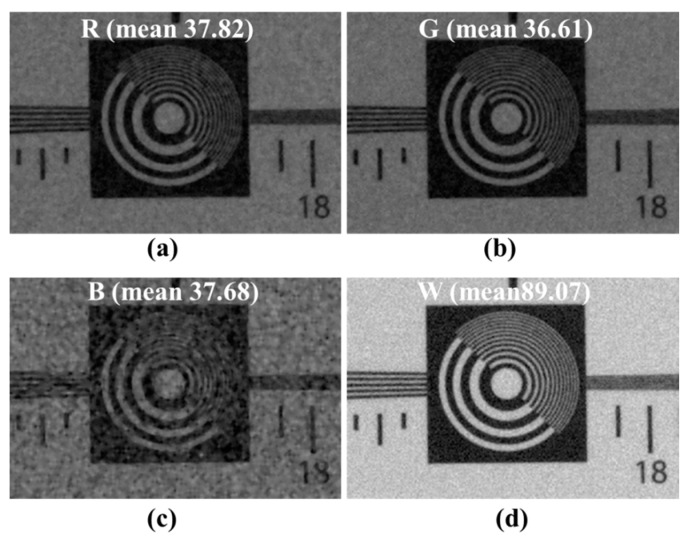
Demosaicing result [24] of the RGBW CFA; (**a**) red (R) channel; (**b**) green (G) channel; (**c**) blue (B) channel; (**d**) white (W) channel.

For Sony RGBW, the ratio of RGB is 50% of Bayer’s RGB area, as shown in Figure 1b. Since the W band includes information of each band of RGB, the correlation between the RGB channels of the W channel is high. Moreover, the W channel has a high ratio of the RGBW CFA. Owing to the high ratio of W, we can obtain high sensitivity and high-resolution images. That is, the W ratio, high sensitivity, and high resolution images can be obtained from the RGBW CFA. However, on account of the high W channel ratio, RGBW CFA demosaicing results in a degraded color resolution. In Figure 2, the image brightness differences of R, G, B, and W channels are compared with the RGBW CFA demosaicing result. The R, G, and B channels are approximately 2.4 times darker than the W channel.

**Figure 3:**
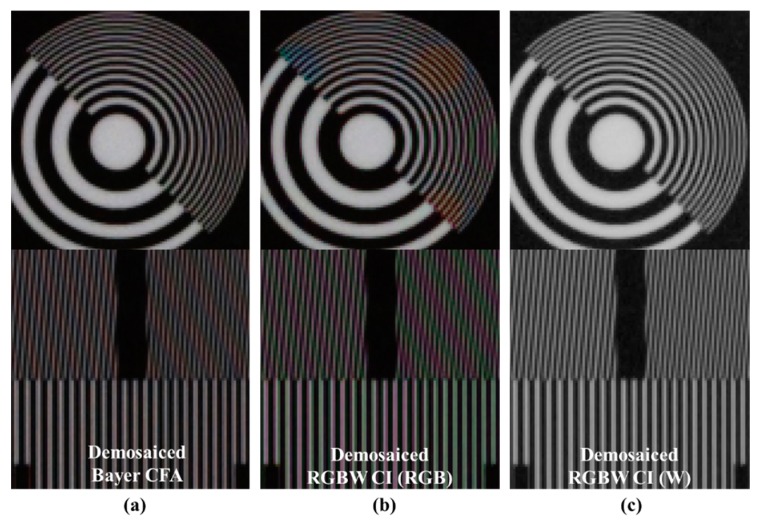
Comparison of Bayer demosaicing and RGBW demosaicing results: (**a**) Bayer CFA demosaicing result [19]; (**b**) red-green-blue (RGB) channels of Sony RGBW CFA demosaicing result [24]; (**c**) W channel of Sony RGBW CFA demosaicing result [24].

Figure 3 compares the Bayer CFA demosaicing and RGBW CFA demosaicing [24] results. In Figure 3a, the red-blue color aliasing artifact occurs in the resolution chart because the ratio of R and B channels is smaller than that of the G channel. In Figure 3b, the RGBW CFA result has more color aliasing than the Bayer CFA result, such as the respective red-blue and green-magenta color aliasing artifacts. Furthermore, each artifact occurs in different directions. Unlike the Bayer CFA, the green-magenta color aliasing artifact occurs in the demosaicing result of the RGBW CFA because the G channel ratio in the RGBW CFA is smaller than in the Bayer CFA. The green-magenta color aliasing artifact of the RGBW CFA occurs in the same direction as the red-blue color aliasing artifact in the Bayer CFA. The green-magenta color aliasing artifact of the RGBW CFA occurs in the same direction as the red-blue color aliasing artifact in the Bayer CFA. In addition, a wider area of red-blue color aliasing artifact occurs. In comparing Figure 3a,c, the spatial resolution of the W channel is similar to the Bayer CFA spatial resolution, which means that the W spatial resolution is not fully reflected in the RGBW demosaicing process.

**Figure 4:**
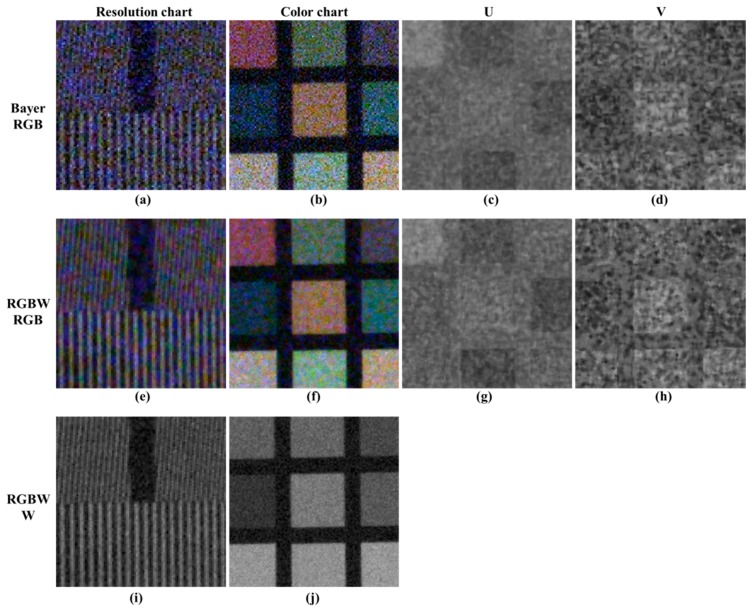
Results of Bayer CFA and RGBW CFA demosaicing in low light conditions: (**a**–**d**) Bayer CFA demosaicing results [19]; (**e**–**h**) RGB channels of Sony RGBW CFA demosaicing results [24]; (**i**,**j**) W channel of Sony RGBW CFA demosaicing results [24].

Figure 4 shows the results of Bayer CFA and RGBW CFA demosaicing in low light conditions for a sensitivity comparison. To compare the sensitivities of W and RGB at the same brightness, a digital gain of 2.4 times (approximately 7.8 dB) is conferred to the RGB channel, except the W channel. In Figure 4a, the separation of the vertical lines due to the noise is inaccurate, and the color of the resolution chart is distorted because of the color noise.

In Figure 4e, the vertical lines of the RGBW CFA demosaicing results are better decomposed than the Bayer CFA demosaicing results, owing to the W channel high sensitivity. However, the color noise energy is still high. Similarly, the effect of the W channel in the color patch slightly reduces the luminance noise energy; however, the color noise energy is similar to the Bayer CFA result. Figure 4c,d,g,h show the U and V channels. The U and V channels are color components of the image in the YUV domain, which are obtained by conversion from the RGB domain. The Bayer CFA and RGBW CFA have similar noise energies. The RGBW color follows that of Bayer, and W improves the resolution restoration ability in the demosaicing process. Nonetheless, the color noise is similar to that of Bayer.

In short, the rationale of the proposed post-processing method is that the reconstructed image has W level brightness and sensitivity. Moreover, the color has a W channel level SNR and color reproduction at the Bayer CFA demosaicing result level. The objective of the proposed algorithm is to address the resolution problem and color aliasing artifacts occurring in the R, G, and B channels by bringing them to the Bayer level using the W channel resolution characteristics. Table 1 shows the characteristics obtained by the proposed post-processing algorithm in the RGBW demosaicing result image. The brightness, sensitivity, and noise characteristics of the image should follow W, and the resolution of W must be followed for the Bayer-level spatial resolution. On the other hand, the hue, saturation, and local contrast must follow the Bayer CFA demosaicing result to maintain color reproduction.

## 3. Proposed Post-Processing Method Based on Texture Decomposition

### 3.1. Post-Processing Framework

To solve the above-described resolution, sensitivity, and color problems in the RGBW CFA demosaicing results, we propose a post-processing method based on texture decomposition. The post-processing approach decomposes the texture components containing noise and resolution information from the image. The sensitivity and resolution of the RGB channels are improved by replacing the W channel texture component with the RGB channel texture. Through the texture decomposer, the image is decomposed into a texture component and a smoothness component. For luminance of image *I*, the texture and smoothness components are expressed as:

(1) $$I=I_{smooth}+I_{texture},$$

where $I_{smooth}$ is the smoothness component of *I*, and $I_{texture}$ is the texture component of *I*. In this paper, we define the texture component as a textural component containing fine-scale details, usually with some periodicity and an oscillatory nature. Accordingly, the texture component is the high-frequency-oriented information; it has the resolution information of the image and the random noise signal. In addition, the texture component contains aliasing artifacts from sampling. The definition of the smoothness component is a component corresponding to the main large object in the image. Therefore, the smoothness component represents the local contrast for color and it has the overall brightness information of the object.

**Figure 5:**
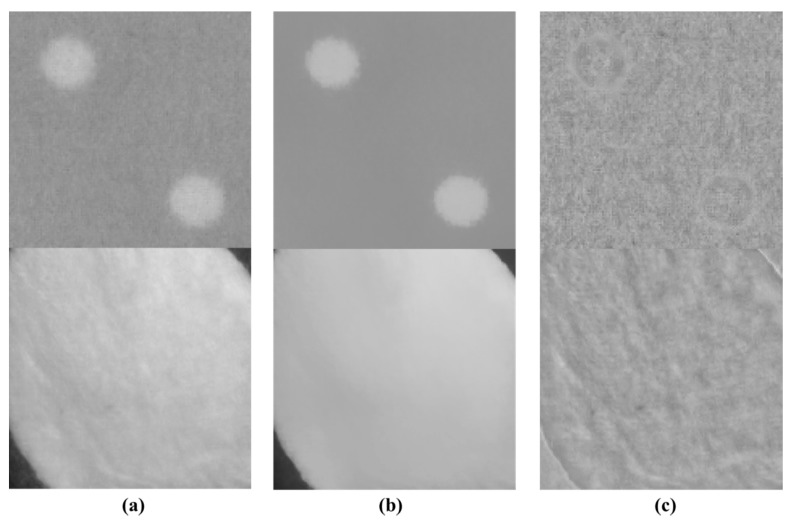
Examples of texture component and smoothness component decomposition: (**a**) Original images; (**b**) smoothness components of original images; (**c**) texture components of original images.

As shown in Table 1, to utilize the W resolution and noise characteristics, a texture component must be decomposed, and a smoothness component must be decomposed to reproduce the RGB color. Figure 5 shows examples of texture and smoothness decomposition of an image. In Figure 5b, the texture decomposer removes only the texture while retaining the edge at the boundary of the polka-dot pattern or white flat region. We hence propose a texture separation method using a smoothing filter. The method of separating texture components through filtering is expressed as:

(2) $$I_{smooth}=I^{filtered},$$

(3) $$I_{texture}=I-I^{filtered},$$

where $I^{filtered}$ is the smoothing filtered result. The proposed filter extracts the smoothness component of the image, as shown in Equation (2), and the filter basically uses a low-pass filter. For the texture components, Equation (3) is derived using Equations (1) and (2). As a result, the texture component is obtained from the difference from the input image as a result of passing the smoothing filter. However, when using a low-pass filter, the smoothness component will lose the strong edges of large objects.

To solve the above problem, we propose a cross multilateral filter, which provides robust classification of color information. The CMF is extended from the cross bilateral filter, which consists of two kernels—a range kernel and a spatial kernel—and three color difference kernels are added. The color difference kernels are added to the CMF to perform accurate filtering in certain areas. The CMF is a smoothing filter applied to the target image by estimating the filter kernel with a single guide image. The CMF is robust to color component edges and it effectively uses color channels, as well as W channels, as guide images to effectively suppress color aliasing and color noise.

**Figure 6:**
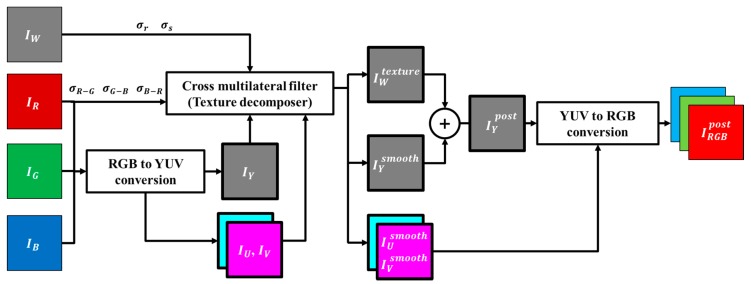
Framework of the proposed post-processing method.

The framework of the proposed algorithm is shown in Figure 6. The proposed post-processing method converts the RGB domain into the brightness and color components of the image so that the RGB color is not changed. The luminance (Y) channel represents the spatial resolution, brightness, and contrast of the image. The U and V channels represent the saturation of the vector with two elements, as well as the hue at the angle. The Y channel extracts the smoothness component through the filter kernel estimated from the W and RGB channels. The smoothness component of the Y channel can retain the color brightness by maintaining the local contrast. It decomposes the texture components of W with the same kernel, which contains the resolution and noise characteristics of the W channel.

Similarly, filtering is performed using the CMF kernel to remove the color aliasing and color noise that are present in the U and V channels. Especially, color aliasing occurs in many texture areas. Therefore, the filtering of the U and V channels can remove the color aliasing of the smoothness component. In addition, CMF using color difference information suppresses the blur occurrence in the object occlusion region of the U and V channels. Finally, the brightness channel of the output image is reconstructed through W texture and Y smoothness.

### 3.2. Texture Component Decomposition with a Cross-Multilateral Filter

A bilateral filter (BF) has two kernels: A range kernel and a spatial kernel. The range kernel represents the distance to pixels in the window for filtering. The spatial kernel represents the difference between the intensity of the pixels in the window. The BF can be expressed as:

(4) $$I_{W}^{BF}\left(p\right)=\frac{1}{W^{BF}\left(p\right)}\underset{q\in S}{\sum }G_{\sigma_{s}}\left(\left|\left|p-q\right|\right|\right)G_{\sigma_{r}}\left(\left|I_{W}\left(p\right)-I_{W}\left(q\right)\right|\right)I_{W}\left(q\right),$$

(5) $$W^{BF}\left(p\right)=\underset{q\in S}{\sum }G_{\sigma_{s}}\left(\left|\left|p-q\right|\right|\right)G_{\sigma_{r}}\left(\left|I_{W}\left(p\right)-I_{W}\left(q\right)\right|\right),$$

where I(p) is the pixel intensity at the p position, $G_{\sigma_{s}}$ and $G_{\sigma_{r}}$ are the Gaussian functions for the spatial and range kernels, respectively, and $\sigma_{s}$ and $\sigma_{r}$ are the smoothing parameters. In addition, p is the current pixel position, S is the window centered in q for the kernel, and q is the pixel position in the S region. Equation (4) represents the BF process of the W channel, $I_{W}^{BF}\left(\cdot \right)$ is the filtered *W* channel, and $I_{W}\left(\cdot \right)$ is the *W* channel. Moreover, $W^{BF}\left(\cdot \right)$ is the normalization term of BF.

Since we must use the same filter kernel for the W channel, as well as the Y, U, and V channels, we use the CBF instead of BF to estimate the kernel on each channel. The CBF can be expressed as:

(6) $$I_{Y|W}^{CBF}\left(p\right)=\frac{1}{W_{W}^{CBF}\left(p\right)}\underset{q\in S}{\sum }G_{\sigma_{s}}\left(\left|\left|p-q\right|\right|\right)G_{\sigma_{r}}\left(\left|I_{W}\left(p\right)-I_{W}\left(q\right)\right|\right)I_{Y}\left(q\right),$$

(7) $$W_{W}^{CBF}\left(p\right)=\underset{q\in S}{\sum }G_{\sigma_{s}}\left(\left|\left|p-q\right|\right|\right)G_{\sigma_{r}}\left(\left|I_{W}\left(p\right)-I_{W}\left(q\right)\right|\right),$$

where $I_{W}$ and $I_{W}$ represent the W and Y channel images, respectively, and $I_{Y|W}^{CBF}\left(\cdot \right)$ is the cross bilateral filtered image of the *Y* channel input by estimating the range kernel from *W*. $W_{W}^{CBF}\left(\cdot \right)$ denotes the normalization term of the CBF estimating the range kernel from *W*. Since the *W* channel has less noise than the *Y* channel, the range kernel is more accurately estimated. However, an error may occur when estimating the range kernel from the *W* channel.

**Figure 7:**
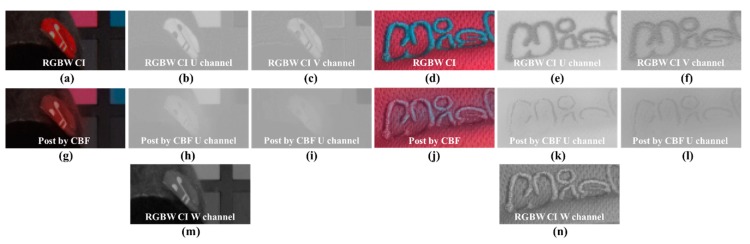
Examples of the kernel estimation error: (**a**,**d**) RGB channels of Sony RGBW CFA demosaicing result [24]; (**b**,**e**) U channel of (**a**,**d**); (**c**,**f**) V channel of (**a**,**d**); (**g**,**j)** RGB channels of post-processing result by CBF; (**h**,**k**) U channel of (**g**,**j**); (**i**,**l**) V channel of (**g**,**j**); (**m**,**n**) W channel of Sony RGBW CFA demosaicing result [24].

Examples of the kernel estimation error are shown in Figure 7. Figure 7g shows a blur in the red circle and gray background. For an exact comparison, Figure 7h,i shows that the circle is blurred, unlike in Figure 7b,c. Furthermore, Figure 7m shows that the intensity difference between the circle and back ground is not considerable. Therefore, in the red circle area, the error of range kernel estimation occurs and the circle is blurred. In the right of Figure 7n, the textures appear to have a minimal difference in brightness. This causes a blur phenomenon, as shown in Figure 7j–l.

In this paper, we therefore propose a CMF that reflects the color difference information in the CBF to solve the above problem. The color difference makes it possible to distinguish different colors for compensating for detection errors in different areas of the color, although the difference in the values is small in the W channel. The expression of CMF is as follows:

(8) $$I_{Y|W}^{CMF}\left(p\right)=\frac{1}{W_{W}^{CBF}\left(p\right)}\underset{q\in S}{\sum }G_{\sigma_{s}}\left(\left|\left|p-q\right|\right|\right)G_{\sigma_{r}}\left(\left|I_{W}\left(p\right)-I_{W}\left(q\right)\right|\right)G_{\sigma_{R-G}}\left(\left|I_{R-G}\left(p\right)-I_{R-G}\left(q\right)\right|\right)G_{\sigma_{G-B}}\left(\left|I_{G-B}\left(p\right)-I_{G-B}\left(q\right)\right|\right)G_{\sigma_{B-R}}\left(\left|I_{B-R}\left(p\right)-I_{B-R}\left(q\right)\right|\right)I_{Y}\left(q\right),$$

(9) $$W_{W}^{CMF}\left(p\right)=\underset{q\in S}{\sum }G_{\sigma_{s}}\left(\left|\left|p-q\right|\right|\right)G_{\sigma_{r}}(\left|I_{W}\left(p\right)-I_{W}\left(q\right)\right|G_{\sigma_{R-G}}\left(\left|I_{R-G}\left(p\right)-I_{R-G}\left(q\right)\right|\right)G_{\sigma_{G-B}}\left(\left|I_{G-B}\left(p\right)-I_{G-B}\left(q\right)\right|\right)G_{\sigma_{B-R}}\left(\left|I_{B-R}\left(p\right)-I_{B-R}\left(q\right)\right|\right),$$

where $I_{Y|W}^{CMF}\left(\cdot \right)$ is the *Y* channel that has passed the estimated CMF from *W* to the range kernel. $W_{W}^{CMF}\left(\cdot \right)$ represents the normalization term of the CMF that estimates the range kernel from *W*. $I_{R-G}\left(\cdot \right)$, $I_{G-B}\left(\cdot \right)$, and $I_{B-R}\left(\cdot \right)$ are the differences between the R, G, and B channels, respectively, and the color difference channels are expressed as:

(10) $$I_{R-G}\left(p\right)=I_{R}\left(p\right)-I_{G}\left(p\right),$$

(11) $$I_{G-B}\left(p\right)=I_{G}\left(p\right)-I_{B}\left(p\right),$$

(12) $$I_{B-R}\left(p\right)=I_{B}\left(p\right)-I_{R}\left(p\right).$$

In Equation (8), the CMF has been extended from the two CBF kernels to five kernels. Three new kernels in the CMF are extended to distinguish areas not distinguishable on the W channel, through color difference channels by color information. Equations (10)–(12) represent the color difference channels.

**Figure 8:**
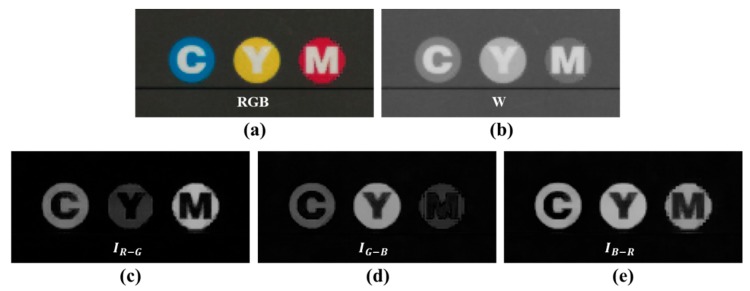
Difference in color separation channels between W and their discrimination ability: (**a**) RGB image; (**b**) W channel image; (**c**) $I_{R-G}$; (**d**) $I_{G-B}$; (**e**) $I_{B-R}$.

The differences among the R, G, and B channels are the color characteristics represented by each band. Owing to the color difference channels, the CMF not only distinguishes the difference in color brightness, but it also distinguishes the areas of local hue and saturation. Figure 8 shows the difference in the color separation channel between W and the discrimination ability. In Figure 8a, there is a circle patch of cyan, yellow, and magenta colors. Figure 8b shows how the W channel represents each circle patch. On a gray background, the “M” circle does not show a large difference in brightness from the background. If the kernel estimate is through CBF, then the range kernel of W will blur the edges of the circle. Figure 8c–e shows the circle patch in the color difference channel. First, the background of the gray color, the letters “C”, “M”, and “Y”, as well as the bottom line, are all black. The color difference channels show that CMF can distinguish between chromatic and achromatic areas. Figure 8c shows that the “M” circle and the “C” circle are well decomposed from the background; however, the “Y” circle shows that the background and letter are not very well decomposed. Similarly, in Figure 8d, the “Y” circle is well decomposed from the background; however, the “M” circle is not well decomposed from the background. In Figure 8e, all circles are well decomposed; however, if they are all in contact, it will be difficult to distinguish between the circle patches with similar degrees of brightness. In Figure 8, to synthesize the color difference channels and the local distinction of the W channels, the AND operation is required for the characteristics detected by the kernel. The color discrimination capabilities of each color difference channel are denoted by the inter-kernel multiplication in (8) for the AND operation.

**Figure 9:**
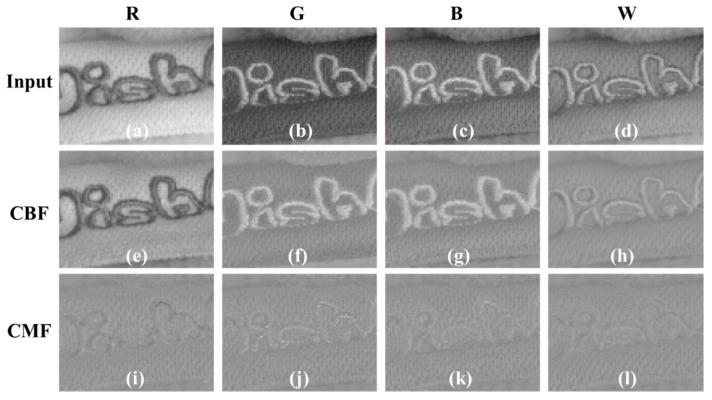
Comparison of texture components decomposed through cross bilateral filter (CBF) and cross multilateral filter (CMF): (**a**–**d**) Original image; (**e**–**h**) texture components of CBF results; (**i**–**l**) texture components of CMF results.

Figure 9 shows a comparison of texture components decomposed through CBF and CMF. In Figure 9a–d, the text in the R, G, B, and W channels is varied in the background. In Figure 9a, the text is darker than the background; in Figure 9b,c, it is brighter than the background. Figure 9d shows the text and background brightness similarly. The result of CBF using only the W channel cannot distinguish between the text and background color, so that all brightness components of the text are expressed in the texture component. The fact that the texture contains the brightness of the text means that the brightness of the text has disappeared in the smoothness component during the filtering process. Figure 9i–l show the texture components decomposed by CMF through the color difference channel. Unlike the results of both the CBF, only texture components, regardless of the brightness difference between the text and the background, are successfully decomposed. To remove the color noise and color aliasing, as well as Y and W channels, the smoothness component of the U and V channels must also be decomposed. The filtering process for the smoothness components of U and V channels can be expressed as follows:

(13) $$I_{U|W}^{CMF}\left(p\right)=\frac{1}{W_{W}^{CBF}\left(p\right)}\underset{q\in S}{\sum }G_{\sigma_{s}}\left(\left|\left|p-q\right|\right|\right)G_{\sigma_{r}}\left(\left|I_{W}\left(p\right)-I_{W}\left(q\right)\right|\right)G_{\sigma_{R-G}}\left(\left|I_{R-G}\left(p\right)-I_{R-G}\left(q\right)\right|\right)G_{\sigma_{G-B}}\left(\left|I_{G-B}\left(p\right)-I_{G-B}\left(q\right)\right|\right)G_{\sigma_{B-R}}\left(\left|I_{B-R}\left(p\right)-I_{B-R}\left(q\right)\right|\right)I_{U}\left(q\right),$$

(14) $$I_{V|W}^{CMF}\left(p\right)=\frac{1}{W_{W}^{CBF}\left(p\right)}\underset{q\in S}{\sum }G_{\sigma_{s}}\left(\left|\left|p-q\right|\right|\right)G_{\sigma_{r}}\left(\left|I_{W}\left(p\right)-I_{W}\left(q\right)\right|\right)G_{\sigma_{R-G}}\left(\left|I_{R-G}\left(p\right)-I_{R-G}\left(q\right)\right|\right)G_{\sigma_{G-B}}\left(\left|I_{G-B}\left(p\right)-I_{G-B}\left(q\right)\right|\right)G_{\sigma_{B-R}}\left(\left|I_{B-R}\left(p\right)-I_{B-R}\left(q\right)\right|\right)I_{V}\left(q\right),$$

where $I_{U|W}^{CMF}\left(\cdot \right)$ and $I_{V|W}^{CMF}\left(\cdot \right)$ are the *U* and *V* channels that have passed the CMF estimated from the range kernel from *W*, respectively.

**Figure 10:**
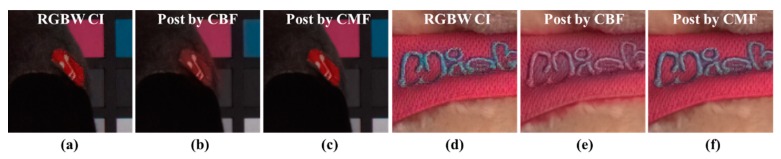
Results of post-processing using CBF and CMF; (**a**,**d**) Sony RGBW CFA demosaicing results [24]; (**b**,**e**) results of post-processing using CBF; (**c**,**f**) results of post-processing using CMF.

Finally, the results of post-processing using CBF and CMF are shown in Figure 10. Figure 10a,d show the demosaicing results of the input RGBW CFA image. Figure 10b,e show the result of post-processing using CBF with a red circle and blurring of the word “Wish”. Figure 10c,f shows the post-processing results using the proposed CMF. The result of the post-processing method using the proposed CMF shows color reproduction that is similar to those in the RGBW CFA demosaicing results.

## 4. Experimental Results

To verify the proposed algorithm, Bayer CFA demosaicing, Sony RGBW CFA demosaicing, and the proposed method post-processing results were compared. We conducted a total of four experiments to assess (1) color aliasing artifacts and spatial resolution; (2) color reproduction; (3) sensitivity and noise in a low light condition; and (4) the SNR and brightness for quantitative comparison. It was shown that the proposed method produced results of good quality in sufficient light conditions. To obtain the original full-resolution R, G, B and W channel images, we used a filter-wheel-installed camera. The wheel contained four different optical filters and was driven by a stepping motor. The four optical filters selectively filtered the R, G, B, and W bands. We captured four photographs of the same scene with the four different filters to obtain the R, G, B, and W channels. Using the four full-resolution channel images, we sampled the R, G, B, and W pixels corresponding to the different patterns, i.e., the Bayer CFA pattern and the Sony RGBW CFA pattern. The photography for the experiment was performed in two light conditions: Normal illumination (1000 lx) and low illumination (1 lx). The normal light condition was used to compare the color aliasing artifacts with spatial resolution and color reproduction. The low light condition was employed to compare the sensitivity, noise, and SNR. To reconstruct the full-color images, demosaicing algorithms suitable for each CFA pattern were applied. In our experiments, the MSG-based demosaicing algorithm from [19] was used for the Bayer pattern. For the Sony RGBW pattern, the MSG-based demosaicing algorithm from [24] was used. The MSG-based method requires no threshold since it makes no hard decisions, and it can be extended to other mosaic patterns.

**Figure 11:**
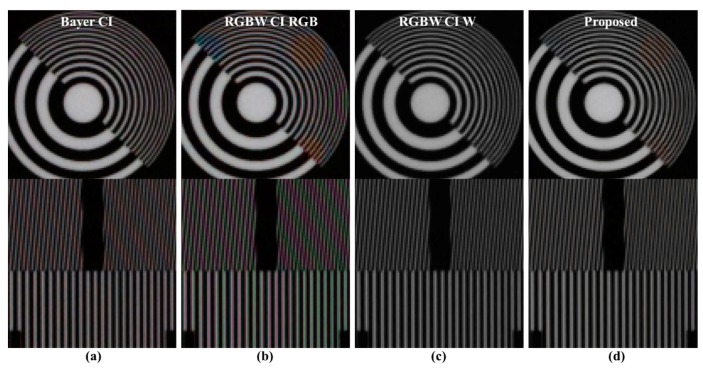
Comparisons of color aliasing artifacts and spatial resolutions: (**a**) Bayer CFA demosaicing results [19]; (**b**) RGB channels of Sony RGBW CFA demosaicing results [24]; (**c**) W channel of Sony RGBW CFA demosaicing results; (**d**) proposed method post-processing results.

Figure 11 depicts comparisons of the color aliasing artifacts and spatial resolutions. Figure 11a shows the Bayer CFA demosaicing result, Figure 11b,c show the Sony RGBW CFA demosaicing result, and Figure 11d shows the result of the proposed post-processing method by entering the images of Figure 11b,c. In the Bayer case, red-blue color aliasing artifacts occur, especially in the longitudinal textures. On the other hand, the result of the RGBW CFA demosaiced RGB channel shows wide diagonal red-blue color aliasing artifacts and green-magenta color aliasing artifacts in the vertical direction. The spatial resolution of the W channel is similar to that of Bayer. The result of the proposed algorithm has Bayer-level spatial resolution. In the vertical texture, the color aliasing artifacts shown in Figure 11b are suppressed. Red-blue color aliasing in the diagonal direction has not completely disappeared; nevertheless, it is significantly suppressed, resulting in better post-processing results than the Bayer CFA demosaicing results.

**Figure 12:**
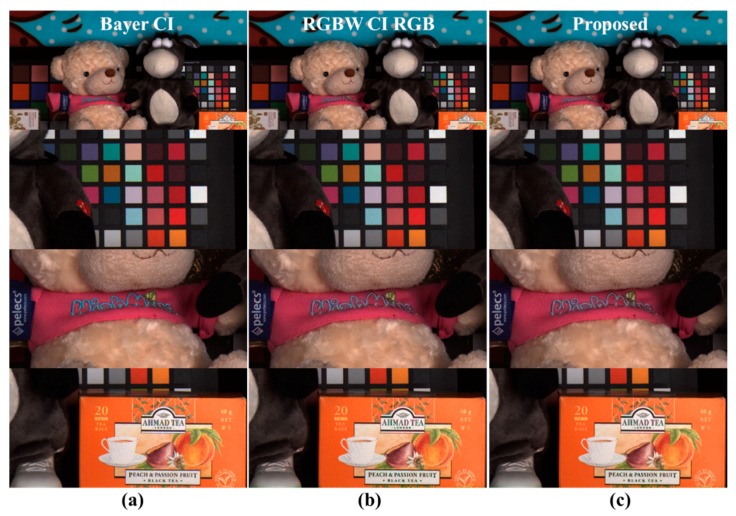
Comparison of color reproduction: (**a**) Bayer CFA demosaicing results [19]; (**b**) RGB channels of Sony RGBW CFA demosaicing results [24]; (**c**) proposed method post-processing results.

The experimental results show that the post-processing of the RGBW CFA demosaicing result has a similar spatial resolution and better color aliasing artifact levels compared to the Bayer CFA demosaicing results. Figure 12 shows the color reproduction comparison. Figure 12a shows the Bayer CFA demosaicing results, Figure 12b presents the Sony RGBW CFA demosaicing results, and Figure 12c depicts the proposed post-processing method results. The first row represents the color of the entire image, all of which represent the color of the Bayer level. The second row shows the color patches, all of which represent Bayer-level colors. The third row shows the dolls, and both the text and background represent the Bayer-level colors. As in Figure 10b,e, Figure 12b shows that the CBF errors are resolved through CMF. The fourth row shows objects with complex and colorful colors, with two results using RGBW CFA (Figure 12b,c) representing the Bayer-level colors. Experimental results demonstrate that the proposed post-processing method results show color reproduction at the Bayer CFA demosaicing result level.

**Figure 13:**
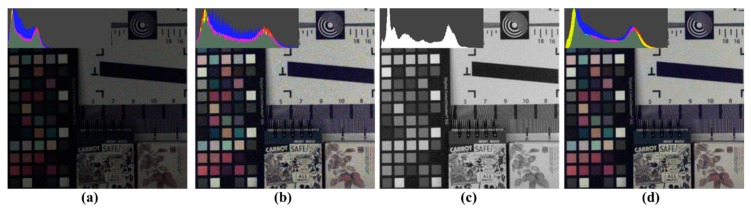
Change in sensitivity depending on the process under a low-light condition: (**a**) RGB channels of Sony RGBW CFA demosaicing results before applying gain [24]; (**b**) RGB channels of Sony RGBW CFA demosaicing results after applying gain [24]; (**c**) W channel of Sony RGBW CFA demosaicing results [24]; (**d**) proposed method post-processing results.

**Figure 14:**
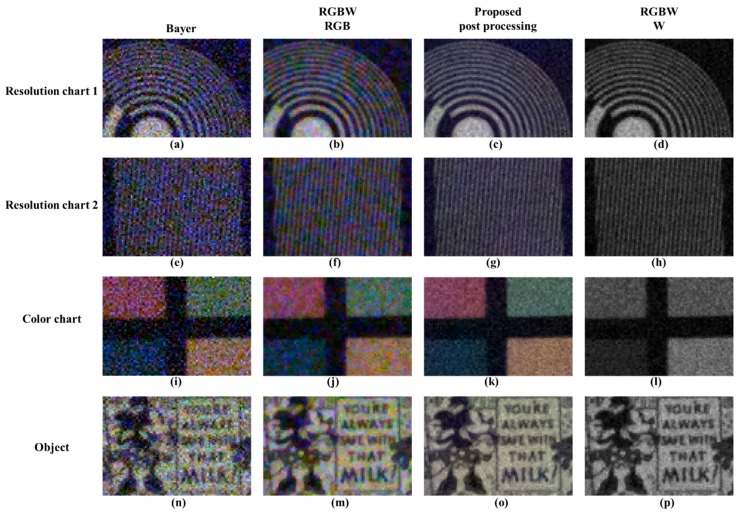
Comparison of luminance / color noise: (**a**,**e**,**i**,**n**) Bayer CFA demosaicing results [19]; (**b**,**f**,**j**,**m**) RGB channels of Sony RGBW CFA demosaicing results [24]; (**c**,**g**,**k**,**o**) proposed post-processing results; (**d**,**h**,**l**,**p**) W channel of Sony RGBW CFA demosaicing results [24].

Figure 13 illustrates the change in sensitivity depending on the process under a low light condition. Figure 13a,c depict the RGBW CFA demosaicing result, which shows the high sensitivity of the W channel compared to the channel-by-channel sensitivity in Figure 2. Figure 13d shows the result of the proposed post-processing method. Figure 13b depicts an image with a 7.8 dB digital gain applied to Figure 13a. In comparing Figure 13b,c, no color shift or washout effect is observed in the color patch. Additionally, by comparing the histograms, it is shown that the post-processing method stably changes the histogram distribution. Through Figure 14, Bayer CFA demosaicing, RGBW CFA demosaicing, and the proposed post-processing approach are compared. The first column is the Bayer CFA demosaicing result, the second column is the Sony RGBW CFA demosaicing result, the third column is the proposed post-processing method result, and the last column is the demosaiced W channel. In the demosaicing results of Bayer CFA and Sony RGB, CFA, RGB, and W are approximately 2.4 times the brightness difference; thus, a 7.8 dB digital gain is applied to each RGB image. When observing the first row and second row, the Bayer CFA demosaicing result has the highest luminance and color noise. The RGBW CFA demosaicing result is better than the Bayer CFA demosaicing result owing to the W effect; however, the color noise is similar to that of Bayer.

**Figure 15:**
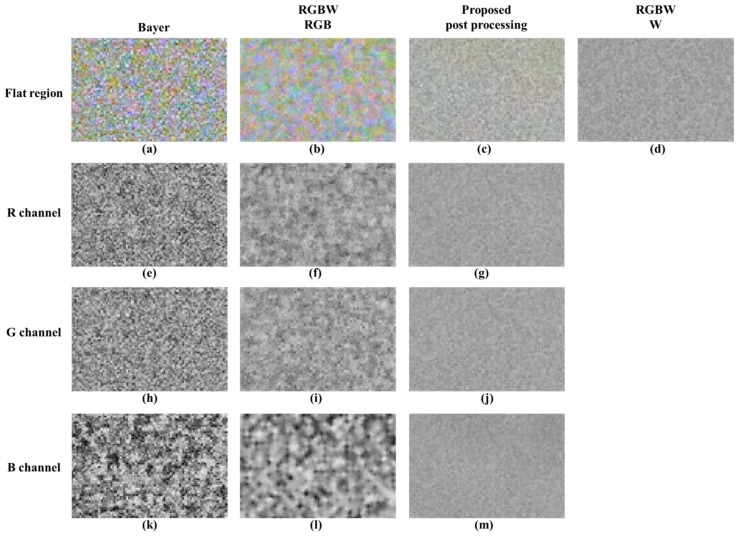
Comparison of luminance / color noise in a flat region: (**a**,**e**,**h**,**k**) Bayer CFA demosaicing results [19]; (**b**,**f**,**i**,**l**) RGB channels of Sony RGBW CFA demosaicing results [24]; (**c**,**g**,**j**,**m**) proposed post-processing results; (**d**) W channel of Sony RGBW CFA demosaicing results [24].

In observing the proposed post-processing method results, all of the luminance color noise is suppressed and the lines that are not decomposed on account of the noise are well represented. In observing the color chart, the proposed post-processing approach reduces both luminance and color noise and makes the color patches uniform in color. Likewise, the proposed post-processing method reduces the luminance and color noise, and it improves the text resolution. Figure 15 shows a comparison of the sensitivity of the flat regions in low light conditions. The proposed method reduces the luminance and color noise. In the R, G, and B channel comparisons, the proposed method improves the image to the noise level of the W channel for R, G, and B channels. In this paper, a quantitative evaluation was performed for improved sensitivity. The basic measuring unit for identifying the quality of an image is the SNR. It is the physical measure of the sensitivity of an imaging system and is identified by applying the 20 log rule [27]. The SNR can be expressed as:

(15) $$SNR_{dB}=20\log_{10}\frac{\mu_{I}}{\sigma_{I}},$$

where $SNR_{dB}$ represents the SNR, and the unit is in decibels (dB) in the log scale. In addition, $\mu_{I}$ is the average of the measurement region in the image, and $\sigma_{I}$ denotes the standard deviation of the measurement region in the image. Table 2 compares the SNR in bright and dark regions at 1 lx. The proposed algorithm has a higher SNR than the Bayer CFA demosaicing results, and it shows an SNR improvement of approximately 8.5 dB in the bright region and 8.5 dB in the dark region. The RGBW demosaicing results also have a higher SNR than Bayer because of *W*, as well as an average SNR improvement of 4 dB in the bright region and an average SNR improvement of 3.9 dB in the dark region. The RGBW demosaicing result and post-processing method result show an average improvement of 4.3 dB in the bright region and an average improvement of 4.6 dB in the dark region.

**Figure 16:**
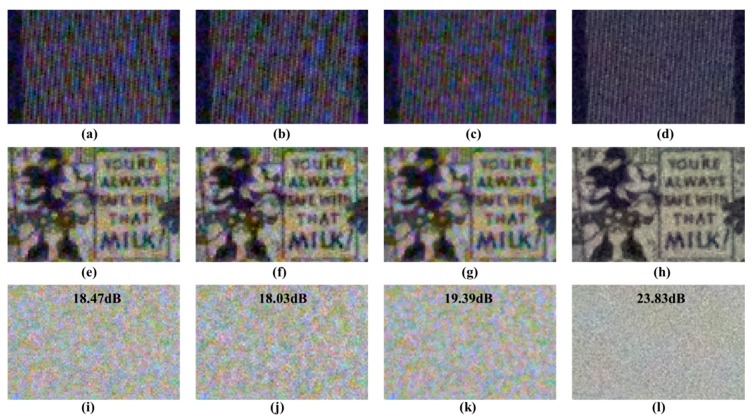
Qualitative and quantitative evaluations comparison of various RGBW demosaicing algorithms: (**a**,**e**,**i**) RGB channels of frequency based demosaicing results [25]; (**b**,**f**,**j**) RGB channels of pan-sharpening based demosaicing results [26]; (**c**,**g**,**k**) RGB channels of multiscale-gradient (MSG)-based demosaicing results [24]; (**d**,**h**,**l**) proposed post-processing results.

Figure 16 shows a comparison of the results of various demosaicing methods for RGBW CFA. The full resolution images are reconstructed using three demosaicing methods: Frequency-based demosaicing algorithm [25], pan-sharpening-based demosaicing algorithm [26], and MSG-based demosaicing algorithm [24]. The top row of Figure 16 shows the resolution chart. The frequency and pan-sharpening-based demosaicing algorithms show more aliasing artifacts than the MSG-based demosaicing algorithm. This finding demonstrates that the MSG-based method using the inter-channel correlation can better restore the high-frequency components than the other two methods. Figure 16 shows a similar level of color noise results from the three demosaicing algorithms. On the other hand, the results of the proposed post-processing show that aliasing artifacts and color noise are significantly reduced. The average SNR value of the RGB channel in the bright flat region is measured, and the proposed method has the highest value.

## 5. Conclusions

In this paper, the RGBW CFA was used to replace the existing Bayer CFA. Moreover, a limit existed in reflecting the W sensitivity through demosaicing. To solve this problem, a post-processing method was proposed in this paper. The proposed algorithm is constructed by reflecting three characteristics: Removal of color aliases and improvement of resolution, W-level SNR and color noise suppression, and effective color reproduction. The texture decomposition method using the proposed CMF is used to reflect the three characteristics. In evaluations, CMF detected not only the texture components, but also the RGBW degradation components, such as noise and color aliasing on RGB channels. At the same time, the image quality was improved by replacing the missing RGB high-frequency components with the texture components estimated from the CMF of the W channel. The experimental results showed that the proposed post-processing approach improved the resolution and color similarity compared to the demosaicing of the Bayer CFA. In addition, the proposed method improved the sensitivity and increased the SNR value by approximately 4.5 dB, by removing noise in low illumination conditions.

## Figures and Tables

**Table 1:** Characteristics of red-green-blue (RGB) and white (W) channels. (o means include, x means not include)

**Characteristics**	**RGB**	**W**
Sensitivity and brightness	x	o
Noise	x	o
Resolution	x	o
Local contrast for color	o	x
Hue & saturation	o	x

**Table 2:** Comparisons of signal-to-noise ratio (SNR) in bright and dark regions at 1 lx.

1 lx	Bright Region			Dark Region		
	Bayer	RGBW	RGBW Post	Bayer	RGBW	RGBW Post
R	16.77	21.72	23.82	4.51	9.26	11.49
G	17.24	21.93	23.64	4.17	9.07	10.98
B	12.72	15.85	24.06	5.40	7.06	16.30
Ave.	15.35	19.39	23.83	4.89	8.75	13.37
